# Identification and genotype–phenotype correlation of *RNF213* p.R4810K and non- p.R4810K variants in Moyamoya disease in an Indian cohort

**DOI:** 10.3389/fneur.2026.1881123

**Published:** 2026-09-17

**Authors:** P. N. Sylaja, Rinta Paul, U. K. Madhusoodanan, Neena S. Kumar, Lakshmi J. Nair, P. Sankara Sarma, Alfiya Fasaludeen, Sunil Kumar, E. R. Jayadevan, Divya Pachat, Sapna Erat Sreedharan, B. Jayanand Sudhir, K. H. Muneeb Hamza, M. V. Anjana Madhu, Moinak Banerjee

**Affiliations:** 1 Department of Neurology, Sree Chitra Tirunal Institute for Medical Sciences and Technology, Thiruvananthapuram, Kerala, India; 2 Department of Biochemistry, Sree Chitra Tirunal Institute for Medical Sciences and Technology, Thiruvananthapuram, Kerala, India; 3 Department of Public Health, Amrita Institute of Medical Sciences and Research Centre, Cochin, Kerala, India; 4 Department of Imaging Sciences and Intervention Radiology, Sree Chitra Tirunal Institute for Medical Sciences and Technology, Thiruvananthapuram, Kerala, India; 5 Department of Clinical Genetics, Aster MIMS, Calicut, Kerala, India; 6 Department of Neurosurgery, Sree Chitra Tirunal Institute for Medical Sciences and Technology, Thiruvananthapuram, Kerala, India; 7 Department of Neurobiology, Rajiv Gandhi Centre for Biotechnology, Thiruvananthapuram, Kerala, India

**Keywords:** genotype–phenotype correlations, Moyamoya disease, RNF213 gene, RNF213 p.R4810K variant, single nucleotide polymorphism

## Abstract

**Background and objectives:**

Moyamoya disease is known to be associated with the Ring Finger 213 gene variant (*RNF213* p.R4810K) in East Asians; however, it remains less studied in South Asians. This study aims to identify the *RNF213* p.R4810K and non- p.R4810K variants and to investigate their phenotypic correlation in an Indian cohort of Moyamoya patients.

**Methods:**

The study prospectively enrolled 100 patients with unilateral or bilateral Moyamoya disease (June 2023 to June 2024). Whole exome sequencing was performed to identify all the *RNF213* exonic variants. The patients were grouped based on the single-nucleotide variants detected (*RNF213* p.R4810K and non- p.R4810K variants) and their pathogenicity. The *RNF213* p.R4810K variants were further categorized as homozygous and heterozygous. The demographic, clinical, and angiographic characteristics were collected. Statistical correlations of *RNF213* polymorphisms with phenotypic features were assessed by Kruskall-Wallis tests and Fisher’s Exact tests.

**Results:**

Seventy-four (74%) patients carried no *RNF213* variants, while 26 (26%) carried *RNF213* variants. The founder variant p.R4810K was detected in 9 (9%) patients, and non- p.R4810K variants in 19 (19%) patients. Five missense variants were reported as novel variants (p.Gln681Glu, p.Pro2875Ser, p.Leu4117Pro, p.Gln4029Arg, p.Asp4003Val). Among these, p.Leu4117Pro and p.Asp4003Val, located within the RING domain, were predicted to destabilize the protein structure. The genotype–phenotype correlation showed that the domicile state (*p* = 0.006), family history of Moyamoya disease (*p* = 0.001), and periventricular anastomosis (*p* = 0.026) were significantly different among patients with p.R4810K and non- p.R4810K variants than those without. There was no significant difference observed in clinical presentation or severity of the disease between homozygous and heterozygous carriers.

**Conclusion:**

The study confirmed that both *RNF213* p.R4810K and non- p.R4810K variants increase the risk of Moyamoya disease with variable phenotypic expression. Further functional studies in a large cohort would help in delineating the role of these variants in disease pathophysiology as well as in better genotype- phenotype correlations.

## 1 Introduction

Moyamoya disease is a rare cerebrovascular disease characterized by progressive stenosis of the distal internal carotid artery and formation of aberrant vascular collateral networks at the base of the brain (1). The clinical manifestations differ between pediatric and adult patients, and may range from asymptomatic to ischemic and hemorrhagic strokes (1). There are geographic differences reported in the annual incidence, age distribution, and clinical presentation of the disease (2–4). Nearly 10–15% of Moyamoya patients have a family history, and the risk of occurrence in family members is approximately 30–40 times higher than in the general population (2). Moreover, ethnic/racial disparities exist in the familial cases; 10–12% in Asian countries versus 2-3% in the United States (3, 5, 6). This indicates a strong influence of genetic predisposition in the pathophysiology of the disease.

Previous genetic studies have identified p.R4810K variant (c.14429G>A, ENST00000582970.1) of the Ring Finger Protein 213 (*RNF213*) gene as a strong susceptibility variant of Moyamoya disease in the East Asian population compared to non-Asians (56% vs. 0%) (7). The recent meta-analysis done in Asians (mostly East Asians included) showed that the earlier disease onset (<18 years), ischemic stroke, familial forms, and posterior cerebral artery involvement were more common in *RNF213* mutants than in wild type (8). Systematic evaluation of non-p.R4810K RNF mutation screening by Moteki et al. (9) in 103 Japanese Moyamoya patients identified a total of 16 missense variants with a minor allele frequency of >1%, but these were not significant after conditioning on the p.R4810K genotype. These findings strongly support the allelic heterogeneity in *RNF213*. The exome sequencing of 28 trios of affected patients and unaffected parents revealed three rare *de novo RNF213* mutants (c.12341C>G, c.12360C>G, c.11986_11989del) associated with childhood onset and involvement of more diffuse occlusive vasculopathy, such as renal artery stenosis (10).

The phenotypic correlation of *RNF213* rare and novel variants has not been investigated in South Asian cohorts. The correlation of these genetic variants with the phenotypic features is crucial in understanding the disease pathophysiology. This study aimed to identify the *RNF213* p.R4810K and non- p.R4810K variants that are likely to impact the idiopathic Moyamoya disease in an Indian cohort and to evaluate the influence of these variants on the demographic, clinical, and angiographic features.

## 2 Methods

### 2.1 Study design and participants

The study prospectively enrolled patients diagnosed with Moyamoya disease between June 2023 and June 2024 from the Comprehensive Stroke Care Program, Department of Neurology, Sree Chitra Tirunal Institute for Medical Sciences and Technology, Thiruvananthapuram, Kerala, India. Patients were included if they were diagnosed with unilateral or bilateral Moyamoya disease, confirmed by digital subtraction angiography, based on the guidelines established by the Research Committee on the pathology and treatment of spontaneous occlusion of the Circle of Willis (11). Patients were excluded if they had (a) known etiology of stroke (large vessel atherosclerotic disease, cardio-embolism, lacunar stroke, or other determined causes); (b) secondary causes of moyamoya disease, such as untreated hyperthyroidism, vasculitis, and hypercoagulable states.

The study was approved by the Institutional Ethics Committee of Sree Chitra Tirunal Institute for Medical Sciences and Technology, Thiruvananthapuram (SCT/IEC/1932/August 2022). Written informed consent was obtained from the participants or their legally authorized representatives. The study was reported in accordance with the Strengthening the Reporting of Genetic Association Studies (STREGA) guidelines (Supplementary material).

### 2.2 Evaluation of clinical and angiographic features

The medical records of all enrolled patients were reviewed to document the demographic and clinical features of patients. The demographic data included age at symptom onset, gender, and domicile state. Patients diagnosed before 18 years of age were considered to have pediatric Moyamoya (12). The following clinical features were captured: the clinical presentation, risk factors, laboratory investigations, initial stroke severity using the National Institutes of Health Stroke Scale (NIHSS) score, functional dependence at the time of admission using the modified Rankin score (mRS), recurrent events at disease onset, treatment received (conservative medical management or revascularization surgery), and peri-procedural events. Family history, including consanguinity, was recorded by directly interviewing the patient and family members.

The angiographic findings were reviewed independently by a neuroradiologist (SK) who was blinded to the clinical and genetic data. The laterality of the vasculopathy (unilateral/bilateral), presence of leptomeningeal collaterals, posterior cerebral artery involvement, presence of the ivy and brush sign, and infarct pattern were assessed. The Suzuki staging was graded from 0 to 6, and the grade was reported on the severely affected hemisphere; scores were categorized as mild (1, 2), moderate (3, 4), and severe (5, 6, 13). The periventricular anastomosis score was defined as the sum of each grade of lenticulostriate artery, thalamic artery, and choroidal artery collaterals for each patient and graded from 0 to 6 (14, 15).

### 2.3 Blood sample collection and DNA extraction

Aseptically drawn 10 mL of peripheral EDTA blood samples were collected from the enrolled patients on the day of enrolment and stored immediately under standard refrigeration conditions. The DNA was extracted using the commercially available QIAamp DNA Blood Midi Kit (QIAGEN). The concentration and purity of the extracted DNA were checked by the microvolume spectrophotometer (Nanodrop, Thermo Scientific). The samples were run on 1% agarose gel electrophoresis to confirm the quality and integrity of the isolated genomic DNA. The DNA samples were labeled using a unique identification number and stored at −80 °C.

### 2.4 Whole-exome sequencing

The exonic regions of the extracted genomic DNAs were sequenced to a mean depth of >80–100X using a targeted next-generation sequencing assay on an Illumina platform at a commercial service provider located in South India. The Genomic Analysis Toolkit (GATK) best practices framework was followed to identify the germline variants in the sample using Sentieon tools. The obtained sequences were aligned to the human reference genome (GRCh38) using the BWA aligner and analyzed. The germline variants identified were annotated using the VariMAT pipeline, and the gene annotation was performed using the Variant Effect Predictor (VEP) program against the Ensembl release 104 human gene model. The previously published variants in literature and a set of databases (ClinVar, OMIM, HGMD, LOVD, DECIPHER, and SwissVar) were accessed to annotate the clinically relevant mutations.

### 2.5 Variant classification

The variants with a population frequency of <0.01% were categorized as rare variants, and the variants that were not previously documented in any of the population databases (1,000 Genomes, IndiaDB, gnomAD, etc.) were categorized as novel variants. The Combined Annotation Dependent Depletion (CADD) tool was used to extract the CADD score to assess the deleteriousness of the single-nucleotide variants reported. Additionally, tools such as Revel, AlphaMissense, PolyPhen-2, SIFT, DANN, MutationTaster and LRT were used to calculate the non-synonymous variants’ effect using the comprehensive interpretation tool by Genoox (Franklin by Qiagen). The clinically relevant variants were included in the data analysis and interpretation of results. The CADD variants were also categorized as benign, likely benign, variant of uncertain significance (VUS), likely pathogenic, and pathogenic based on the American College of Medical Genetics and Genomics (ACMG) guidelines. A multiple sequence alignment was performed using Clustal Omega to determine the amino acid conservation of the variants across the species (16).

### 2.6 Genotype–phenotype correlation analysis

To analyze the phenotypic correlation with the *RNF213* genotypes, patients were categorized into three groups based on the variants detected: (i) patients without *RNF213* variants, (ii) patients with *RNF213* p.R4810K variants, and (iii) patients with *RNF213* non- p.R4810K variants. Patients with compound heterozygous variants were excluded from the analysis. Parental carrier testing was not done for these patients to determine the contribution of each variant in a compound heterozygous state. Patients with *RNF213* p.R4810K variants were further grouped as homozygous (AA) and heterozygous (GA) and compared with the *RNF213* genotype (GG). Each category was compared against the demographic, clinical, and angiographic characteristics and validated by statistical scores.

### 2.7 Statistical analysis

Data entry was done in a Microsoft Excel spreadsheet, and statistical analysis was done using STATA Basic Edition version 18 (StataCorp, Texas, United States). Correction for multiple comparisons by the Benjamini-Hochberg method was done using an online tool available at https://www.sdmproject.com/utilities. Numeric variables were summarized as median (Interquartile Range). Categorical variables were presented as frequencies and percentages. Statistical significance of the associations of the background characteristics and phenotypic features with *RNF* variants was assessed by Kruskal-Wallis tests for numeric variables since they were not normally distributed (based on Shapiro–Wilk’s tests) and Fisher’s Exact tests for categorical variables. *p*-values <0.05 were considered to conclude statistical significance. Due to small sample sizes, the bivariate statistical analysis was based on Exact tests, and there was no scope to attempt any multivariable analysis.

## 3 Results

### 3.1 Demographic, clinical, and angiographic characteristics

A total of 100 patients were enrolled in the study. The flow chart of the patient selection and enrollment is shown in Figure 1. The baseline characteristics of the enrolled patients are summarized in Table 1. The mean age at onset of symptoms was 16.14 ± 14.64 years in pediatric patients and 30.31 ± 11.72 years in adult patients. The vasculopathy was bilateral in 87 (87%) patients. The most common clinical presentation was ischemic stroke (59%), and the most common risk factor was hypertension (14%). Family history was reported in 4 (4%) patients (Table 1, Supplementary Figure 1). There were recurrent events in 62 (62%) patients at disease onset. The posterior cerebral artery was involved in 19 (19%) patients. Moderate-to-severe Suzuki staging (stages 3, 4, and 5) was seen in 86 (86%) patients. Periventricular anastomosis was present in 93 (93%) patients.

**Figure 1:**
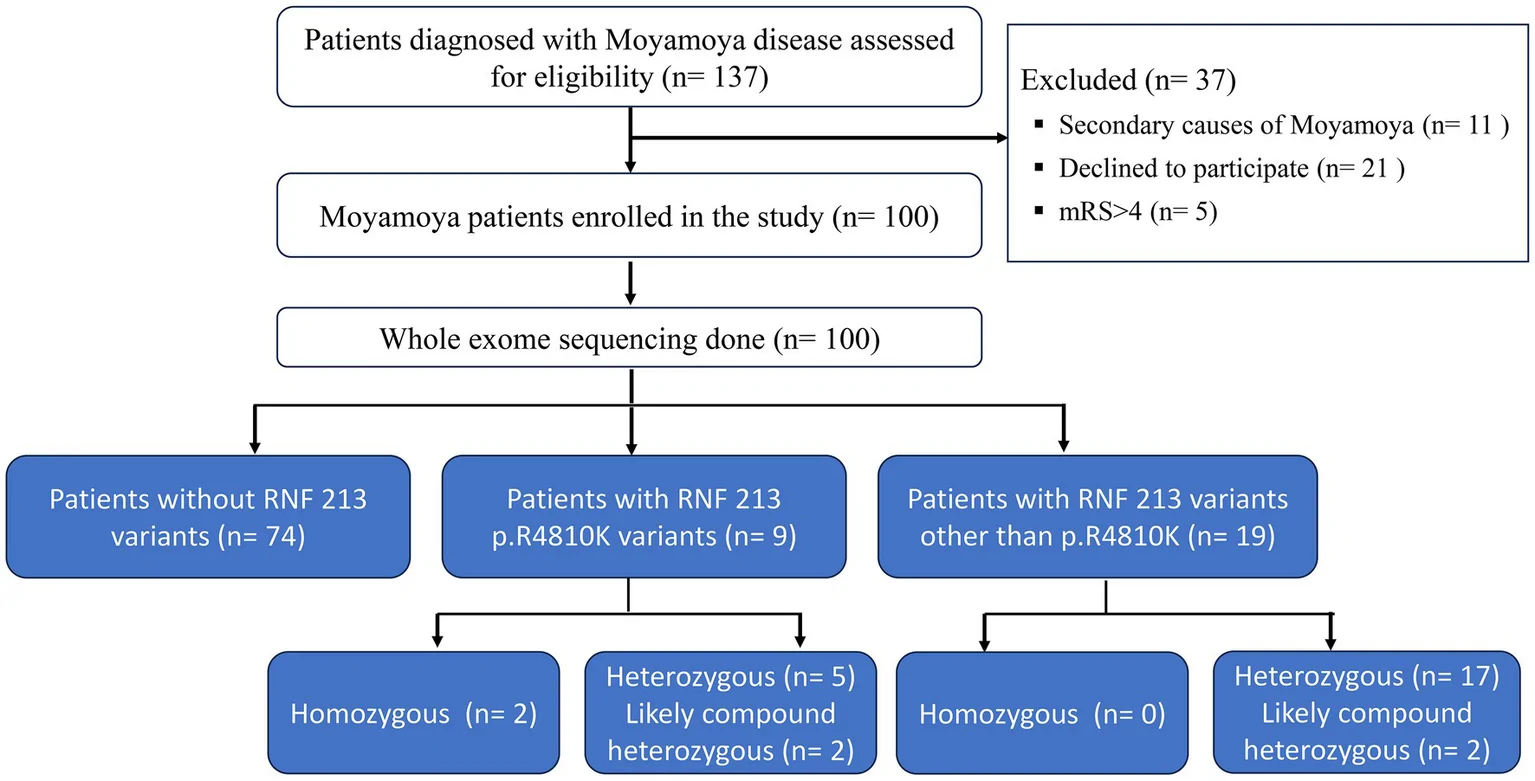
Flow diagram of the patient enrollment and genotyping.

**Table 1:** Baseline characteristics of the patients with Moyamoya disease.

Baseline characteristics	*n* = 100
Demographic characteristics	
Age at symptoms onset (years), mean ± SD	
Pediatric	16.14 ± 14.64
Adults	30.31 ± 11.72
Age at symptoms onset (years), *n* (%)	
<18 years	61 (61%)
≥18 years	39 (39%)
Gender, *n* (%)	
Male	47 (47%)
Female	53 (53%)
Domicile state, *n* (%)	
Kerala	35 (35%)
Tamil Nadu	46 (46%)
West Bengal	7 (7%)
Others	12 (12%)
Clinical characteristics	
Initial clinical presentation, *n* (%)	
Transient ischemic attack	40 (40%)
Ischemic stroke	59 (59%)
Hemorrhage	11 (11%)
Headache	16 (16%)
Seizure	35 (35%)
Symptoms, *n* (%)	
Weakness	75 (75%)
Speech disturbances	42 (42%)
Seizures	44 (44%)
Headache	30 (30%)
Others	13 (13%)
Laterality, *n* (%)	
Unilateral	13 (13%)
Bilateral	87 (87%)
Risk factors, *n* (%)	
Hypertension	14 (14%)
History of thyroid disease	9 (9%)
Consanguinity	9 (9%)
Current smoking	6 (6%)
Alcohol intake	4 (4%)
Diabetes	3 (3%)
Hyperlipidaemia	1 (1%)
Others	7 (7%)
Family history of Moyamoya disease, *n* (%)	4 (4%)
NIHSS score at enrolment, *n* (%)	
0	66 (66%)
1–4	28 (28%)
5–15	6 (6%)
mRS score at enrolment, *n* (%)	
Favorable (≤2)	87 (87%)
Unfavorable (>2)	13 (13%)
Recurrent events at disease onset, *n* (%)	62 (62%)
Treatment, *n* (%)	
Conservative medical management	14 (14%)
Revascularisation	86 (86%)
Periprocedural events, *n* (%)	8 (9.3%)
Imaging characteristics	
Posterior cerebral artery involvement, *n* (%)	19 (19%)
Leptomeningeal collaterals present, *n* (%)	98 (98%)
Suzuki staging, *n* (%)	
Stages 1-2	14 (14%)
Stage 3–6	86 (86%)
Ivy sign present, *n* (%)	38 (38%)
Brush sign present, *n* (%)	13 (13%)
Periventricular anastomosis score, *n* (%)	
0	5 (5%)
1	20 (20%)
2	24 (24%)
3	19 (19%)
4	16 (16%)
5	8 (8%)
6	6 (6%)
Infarct pattern on the right hemisphere, *n* (%)	
Watershed	25 (25%)
Cortical–subcortical	37 (37%)
Hemorrhage	4 (4%)
Infarct pattern on the left hemisphere, *n* (%)	
Watershed	34 (34%)
Cortical–subcortical	33 (33%)
Hemorrhage	1 (1%)

SD, standard deviation; NIHSS, National Institutes of Health Stroke Scale; mRS, modified Rankin Scale.

### 3.2 Identification of *RNF213* p.R4810K and non- p.R4810K variants

The *RNF213* genotyping data were extracted from the whole-exome sequencing data. Out of 100 Moyamoya disease probands, 26 (26%) patients carried *RNF213* missense variants, and 74 patients carried no *RNF213* variants. There were no frameshift or nonsense mutations found. The NGS-based CNV assay did not identify any larger exonic gene rearrangements as well. Among the 26 patients with *RNF213* variants, 9 had the *RNF213* p.R4810K variant, and 19 had non-p.R4810K variants. Two patients had likely compound heterozygous variants involving both RNF213 p.R4810K and non- p.R4810K variants. Figure 2 depicts the schematic representation of the RNF213 protein and variants identified. The description of *RNF213* variants, including variant type, zygosity, allele frequency, and in silico prediction scores, is provided in Supplementary Table 1. The variants were evolutionarily conserved across species (Supplementary Table 1, Supplementary Figure 2).

**Figure 2:**
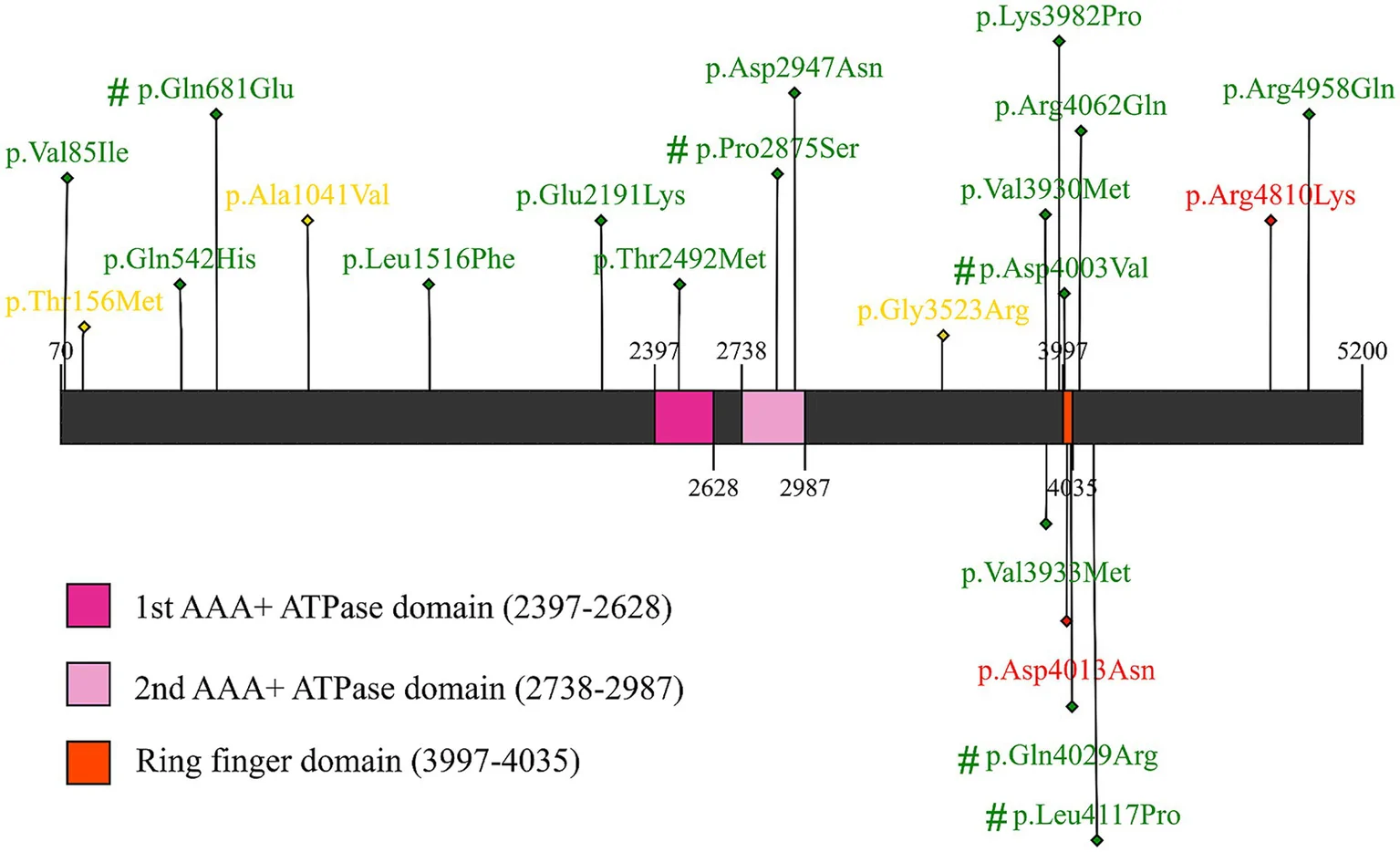
Schematic representation of the RNF213 protein. The likely pathogenic variants are shown in red, benign or likely benign in yellow, and variant of uncertain significance in green. #Novel variants The image was created using the Illustrator for Biological Sciences version 2.0 (IBS 2.0): An upgraded illustrator for the visualization of biological sequences. Nucleic Acids Research, 2022. DOI: 10.1093/nar/gkac373.

The *RNF213* p.R4810K variant showed a population frequency of 0.03 and 0.02% in gnomAD-Genome and gnomAD-Exome databases, respectively. The *in-silico* predictions of the variant were benign based on Revel, AlphaMissense, and SIFT databases, but predicted to be deleterious based on the DANN database. Although, as per ACMG guidelines, classification of this variant is likely pathogenic, cumulative clinical evidence identifies *RNF213* p.R4810K as a pathogenic variant of Moyamoya disease (Clinvar and Uniprot classification).

Among 19 patients, a total of 20 non-p.R4810K variants were observed. Two variants, p.Ala1041Val and p.Thr2492Met, had a population frequency of 0.02% in the gnomAD-Genome database. Five variants were identified as novel variants that include p.Gln681Glu, p.Pro2875Ser, p.Leu4117Pro, p.Gln4029Arg, and p.Asp4003Val, and 13 variants were found to be rare variants (Supplementary Table 1). The cryo-electron microscopy structure of human RNF213 (PDB ID: 9JW1) was downloaded from the Protein Data Bank (PDB) and used for structural analysis. The five novel variants were mapped onto the RNF213 structure and visualized using PyMOL v3.1.8. (Supplementary Figure 3). The structural stability effect of each variant was assessed by estimating the change in protein stability (ΔΔ*G*, total energy) using FoldX v5.1. The protein structure was repaired using the RepairPDB function in FoldX, and the ΔΔ*G* values for the novel variants were calculated using the BuildModel command. The predicted structural effects of the variants are summarized in Supplementary Table 2.

The non-p.R4810K variants were classified as likely pathogenic (p.Asp4013Asn), benign (p.Thr156Met, p.Gly3523Arg), likely benign (p.Ala1041Val), and others as VUS (Supplementary Table 1). The amino acid sequences of these variants were conserved across the species, which further signifies the essential role of these variants in the protein function and pathogenicity.

Among the 26 *RNF213* variants, eleven variants (p.Glu2191Lys, p.Asp4013Asn, p.Leu1516Phe, p.Val3930Met, p.Lys3982Arg, p.Val3933Met, p.Leu4117Pro, p.Arg4062Gln, p.Asp4003Val, p.Thr2492Met, p.Asp2947Asn) with a CADD score greater than 20 were considered to be damaging (Supplementary Table 1). The RNF213 variant spectrum and the associated phenotypic features differ across East Asian, South-East Asian, and Caucasians and have been summarized in Supplementary Table 3. In addition to RNF213, we interrogated other genes associated with Moyamoya disease, such as *GUCY1A1*, *ZNF783*, *ZXDC* (Supplementary Table 4).

### 3.3 Genotype–phenotype correlation of *RNF213* variants vs. the wild genotype

The comparison of phenotypic features of *RNF213* p.R4810K and non- p.R4810K variants with the wild genotype is shown in Table 2. Patients with *RNF213* p.R4810K variants were mostly resided in north-west states, especially West Bengal, compared to southern regions (71.4% vs. 28.6%, *p* = 0.006), had a positive family history of Moyamoya disease (*p* = 0.001), and had a greater score of periventricular anastomoses (*p* = 0.026). Patients with non- p.R4810K variants were from southern states, especially Kerala and Tamil Nadu (88.2% vs. 11.2%, *p* = 0.006).

**Table 2:** Comparison of phenotypic characteristics among *RNF213* p.R4810K and non- p.R4810K variants with the *RNF213* wild genotype.

Characteristics	Patients without *RNF 213* variants (*n* = 74)	Patients with *RNF 213* p.R4810K variants (*n* = 7)	Patients with *RNF 213* non-p.R4810K variants (*n* = 17)	*p*-value^#^
Age at symptoms onset (years), median (IQR)	21.0 (28.0)	21.0 (47.1)	17.0 (23.0)	0.236
Gender, *n* (%)				
Male	32 (43.2%)	2 (28.6%)	12 (70.6%)	0.117
Female	42 (56.8%)	5 (71.4%)	5 (29.4%)	
Domicile state, *n* (%)				
Kerala	29 (39.2%)	0 (0)	6 (35.3%)	**0.006**
Tamil Nadu	35 (47.3%)	2 (28.6%)	9 (52.9%)	
West Bengal	0 (0)	5 (71.4%)	1 (5.9%)	
Madhya Pradesh	2 (2.7%)	0 (0)	0 (0)	
Andhra Pradesh	2 (2.7%)	0 (0)	0 (0)	
Maharashtra	2 (2.7%)	0 (0)	0 (0)	
Gujarat	1 (1.4%)	0 (0)	0 (0)	
Rajasthan	1 (1.4%)	0 (0)	1 (5.9%)	
Andaman	1 (1.4%)	0 (0)	0 (0)	
Uttar Pradesh	1 (1.4%)	0 (0)	0 (0)	
Domicile state, *n* (%)				
South Indian states (Kerala, Tamil Nadu, Andhra Pradesh)	66 (89.2%)	2 (28.6%)	15 (88.2%)	**0.006**
North, west and east states	8 (10.8%)	5 (71.4%)	2 (11.8%)	
Initial clinical presentation as seizure, *n* (%)				
No	54 (73%)	3 (42.9%)	8 (47.1%)	0.092
Yes	20 (27%)	4 (57.1%)	9 (52.9%)	
Family history of Moyamoya disease, *n* (%)				
No	74 (100%)	3 (42.9%)	17 (100%)	**0.001**
Yes	0 (0)	4 (57.1%)	0 (0)	
Consanguinity				
No	68 (91.9%)	6 (85.7%)	17 (100%)	0.395
Yes	6 (8.1%)	1 (14.3%)	0 (0)	
Platelet count, median (IQR)	2.8 (1.1)	1.7 (0.7)	2.6 (1.1)	**0.026**
Posterior cerebral artery involvement, *n* (%)				
Absent	59 (80.8%)	4 (57.1%)	16 (94.1%)	0.133
Present	14 (19.2%)	3 (42.9%)	1 (5.9%)	
Leptomeningeal collaterals, *n* (%)				
Absent	1	0	0	1.000
Present	72 (98.6%)	7 (100%)	17 (100%)	
Suzuki staging, *n* (%)				
Stages 1-2	9 (12.3%)	0 (0)	4 (23.5%)	0.3949
Stages 3–6	64 (87.7%)	7 (100%)	13 (76.5%)	
Periventricular anastomosis, *n* (%)				
0	4 (5.4%)	1 (14.3%)	0 (0)	**0.0264**
1	16 (21.6%)	0 (0)	4 (23.5%)	
2	18 (24.3%)	0 (0)	5 (29.4%)	
3	17 (23%)	1 (14.3%)	0 (0)	
4	10 (13.5%)	1 (14.3%)	5 (29.4%)	
5	5 (6.8%)	1 (14.3%)	2 (11.8%)	
6	2 (2.7%)	3 (42.9%)	1 (5.9%)	

Two patients with RNF 213 variants (compound heterozygotes) were excluded from this table and analysis. Sensitivity analysis was performed by including and excluding the compound heterozygotes. Patients without RNF 213 variants (*n* = 74) were compared with the RNF 213 variants group (*n* = 24) while excluding the 2 compound heterozygotes, and also compared with the RNF 213 variants group (*n* = 26) while including the 2 compound heterozygotes. Statistical significance at the 5% level did not differ for any of the variables in Table 2. RNF213, Ring Finger 213, SD, standard deviation; IQR, interquartile range. ^#^Benjamini-Hochberg procedure. Bold values represent *p* < 0.05 indicating statistical significance.

### 3.4 Genotype–phenotype correlation of homozygous and heterozygous *RNF213* p.4810K variants

The comparison between homozygous and heterozygous *RNF213* p.R4810K variants with the wild type showed that the homozygous variants were present in patients who resided in Tamil Nadu and West Bengal (*p* = 0.0006). We did not observe any significant difference in the clinical presentation, angiographic features or the Suzuki staging between the groups (Table 3).

**Table 3:** Comparison of phenotypic characteristics among *RNF213* p.R4810K homozygous and heterozygous variants with the *RNF213* p.R4810K wild genotype.

Characteristics	GG (*n* = 91)	GA (*n* = 7)	AA (*n* = 2)	*p*-value^#^
Age at symptoms onset (years), median (IQR)	18.5 (25.8)	36.0 (52.8)	5.5 (1.0)*	0.142
Gender, *n* (%)				
Male	44 (48.4%)	2 (28.6%)	1 (50%)	0.779
Female	47 (51.6%)	5 (71.4%)	1 (50%)	
Domicile state, *n* (%)				
Kerala	35 (38.5%)	0 (0)	0 (0)	**0.0006**
Tamil Nadu	44 (48.4%)	1 (14.3%)	1 (50%)	
West Bengal	1 (1.1%)	5 (71.4%)	1 (50%)	
Madhya Pradesh	2 (2.2%)	1 (14.3)	0 (0)	
Andhra Pradesh	2 (2.2%)	0 (0)	0 (0)	
Maharashtra	2 (2.2%)	0 (0)	0 (0)	
Gujarat	1 (1.1%)	0 (0)	0 (0)	
Rajasthan	2 (2.2%)	0 (0)	0 (0)	
Andaman	1 (1.1%)	0 (0)	0 (0)	
Uttar Pradesh	1 (1.1%)	0 (0)	0 (0)	
Domicile state grouping, *n* (%)				
South Indian states (Kerala, Tamil Nadu, Andhra Pradesh)	81 (89%)	1 (14.3%)	1 (50%)	**0.001**
North, west and east states	10 (11%)	6 (85.7%)	1 (50%)	
Initial clinical presentation as seizure, *n* (%)				
No	62 (68.1%)	2 (28.6%)	1 (50%)	0.110
Yes	29 (31.9%)	5 (71.4%)	1 (50%)	
Symptoms- headache, *n* (%)				
No	66 (72.5%)	2 (28.6%)	2 (100%)	0.072
Yes	25 (27.5%)	5 (71.4%)	0 (0)	
Family history of Moyamoya, *n* (%)				
No	91 (100%)	4 (57.1%)	1 (50%)	**0.001**
Yes	0 (0)	3 (42.9%)	1 (50%)	
Triglycerides, median (IQR)	87.5 (62.3)	113.0 (86.0)	208.0 (16.0)*	0.205
Posterior cerebral artery involvement, *n* (%)				
Absent	75 (83.3%)	5 (71.4%)	0 (0)	0.067
Present	15 (16.7%)	2 (28.6%)	2 (100%)	
Leptomeningeal collaterals, *n* (%)				
Absent	1 (1.1%)	0 (0)	0 (0)	1.000
Present	89 (98.9%)	7 (100%)	2 (100%)	
Suzuki staging, *n* (%)				
Stages 1-2	13 (14.4%)	0 (0)	0 (0)	0.779
Stages 3–6	77 (85.6%)	7 (100%)	2 (100%)	
Brush sign, *n* (%)				
No	77 (84.6%)	6 (85.7%)	0 (0)	0.052
Yes	11 (12.1)	0 (0)	2 (100%)	
Periventricular anastomosis, *n* (%)				
0	4 (4.4%)	1 (14.3%)	0 (0)	0.067
1	20 (22%)	0 (14.3%)	0 (0)	
2	23 (25.3%)	1 (14.3%)	0 (0)	
3	17 (18.7%)	2 (28.6%)	0 (0)	
4	15 (16.5%)	1 (14.3%)	0 (0)	
5	7 (7.7%)	1 (14.3%)	0 (0)	
6	3 (3.3%)	1 (14.3%)	2 (100%)	

RNF, Ring Finger Protein; GG, wild type; GA, heterozygous; AA, homozygous. IQR, interquartile range. *Range (maximum–minimum) instead of IQR due to small sample sizes. ^#^Benjamini-Hochberg procedure. Bold values represent *p* < 0.05 indicating statistical significance.

### 3.5 Comparison of phenotypic features among *RNF213* variants based on pathogenicity

Among the *RNF213* variants analyzed, two variants (p.Arg4810Lys, p.Asp4013Asn) identified in eight patients were classified as likely pathogenic, two variants (p.Thr156Met, p.Ala1041Val) as benign or likely benign in three patients, and others as VUS. Two patients with compound heterozygosity were excluded from the analysis. The benign or likely benign variants were grouped under VUS for the statistical analysis. The comparison among VUS and likely pathogenic variants with the wild type showed that the domicile state (*p* = 0.012), positive family history (*p* = 0.0002), and periventricular anastomosis (*p* = 0.019) were significantly different among the groups (Supplementary Table 5).

The small subgroup sizes, especially the homozygous p.R4810K carriers, lead to a low statistical power; hence, all the significant findings reported in the study are exploratory.

## 4 Discussion

In this study, we have identified the *RNF213* p.R4810K and non- p.R4810K variants in Moyamoya patients of Indian origin and described their correlation with both clinical and angiographic characteristics.

The present study has identified both homozygous and heterozygous *RNF213* p.R4810K genotypes as a strong pathogenic variant of Moyamoya disease in 26 (26%) patients. The founder variant of Moyamoya disease, *RNF213* p.R4810K, has been reported to be higher in East Asians, especially among Japanese (70–90%) and Korean patients (60–80%) (7); however, it is rare or absent in Southeast Asians and Caucasians (17–19). European/American cohorts showed a broad spectrum of rare RNF213 variants and additional susceptibility genes (19). Our preliminary study of 65 Indian Moyamoya patients identified the homozygous RNF213 p.R4810K variant in 5 (8%) patients (5). There is no large cohort data available from South Asians. Similar to Caucasian data, the RNF213 p.R4810K variant was less common in our Indian cohort, while several rare missense RNF213 variants were present. These findings elucidate a regional disparity of *RNF213* p.R4810K genotypic expression among South Asians.

This is the first report from India investigating the role of *RNF213* non- p.R4810K variants in Moyamoya patients. The study identified five novel *RNF13* variants (Figure 2), three of them (p.Leu4117Pro, p.Gln4029Arg, p.Asp4003Val) were clustered around the C-terminal region of the RNF213 protein harboring the RING finger domain, one (p.Pro2875Ser) was in the AAA + ATPase domain, and one (p.Gln681Glu) was in the N-terminal region. The missense variants in the RING finger domain may have deleterious effects on the protein’s E3 ubiquitin ligase activity, while the variants in the AAA + ATPase domains are primarily involved in the regulation of protein function (20). Takeda et al. reported that the disruption of E3 ubiquitin ligase activity might affect the nuclear factor kB (NF-KB) and apoptosis-inducing function of RNF213 and contribute to the Moyamoya disease pathogenesis (21). The recent functional studies have expanded the role of RNF213 in diverse biological functions such as regulation of lipid droplet dynamics, inflammation-related angiogenesis, and antimicrobial defense, suggesting that disruption of these functions may contribute to disease pathogenesis (22) (23). The structural mapping of novel variants showed that the variants L4117P and D4003V, located within the RING domain, were predicted to destabilize the protein structure, with L4117P causing stronger destabilization. P2875S, located in the second AAA + ATPase domain, is predicted to have moderately destabilizing effects on the protein structure. The conclusive findings on these variants are warranted, as further variable threshold tests need to be done and validated in large case–control studies. The functional impacts of novel variants are only hypotheses based on in silico prediction and protein domain location, which emphasizes that further functional experiments are required to validate the findings.

There were 13 rare variants identified, three of them (p.Asp4013Asn, p.Arg4062Gln, p.Val3933Met) were found to have previously been described in both East Asians (China) and Caucasians (7, 19, 24, 25). Interestingly, the *RNF213* p.Asp4013Asn located in the RING finger domain was found to be a pathogenic and deleterious variant of Moyamoya disease. The p.Asp4013Asn showed incomplete penetrance in previous studies, suggesting the necessity of additional environmental or genetic factors for developing Moyamoya disease (7). The functional analysis of p.Asp4013Asn showed its inhibitory effects on re-endothelization and alteration of angiogenesis (26). The evolutionary conservation of amino acid regions of these variants further suggests that alterations at these positions were likely to disrupt protein structure or function and may therefore contribute to pathogenicity.

The pathological roles of *RNF213* polymorphism in Moyamoya disease are yet to be explored, even though the previous evidence suggests the role of *RNF213* in the regulation of gene expression of vascular endothelial cells (27) and in the modulation of vascular tortuosity of the internal carotid artery (28). We found that the clinical and angiographic features were significantly different among the patients expressing *RNF213* p.R4810K and non- p.R4810K variants. The *RNF213* p.R4810K variant was present in all familial cases reported in our cohort, while the non-p.R4810K variants were present only in sporadic Moyamoya cases. The correlation of positive family history and the *RNF213* p.R4810K is already known from the previous studies (29–31), and this would recommend the genetic screening among family members to rule out the asymptomatic Moyamoya cases. The pedigrees do reflect incomplete penetrance of the RNF213 p.R4810K. We could not perform the genotyping of the unaffected family members to measure the appropriateness of penetrance based on genotypes. The observed skipping of generations and the presence of unaffected obligate carriers in Families A and B are consistent with autosomal-dominant inheritance with incomplete penetrance. Although p.R4810K is the strongest known genetic susceptibility variant for moyamoya disease in East Asian populations, epidemiological studies estimate that only approximately 1.7–2.7% of carriers develop moyamoya disease, corresponding to an estimated penetrance of approximately 1 in 150–300 carriers in East Asian populations, as reported by Liu et al. (32) and Ohara et al. (33). This indicates that the variant is considered a susceptibility factor rather than a fully disease-causing variant, with reduced penetrance.

We observed a significant regional disparity in the expression of *RNF213* variants in our study. Patients who resided in the north eastern states of India, especially West Bengal, carried *RNF213* p.R4810K variants, whereas patients who resided in South Indian regions carried *RNF213* non- p.R4810K variants (71.4% vs. 11.8%). This genomic variation could be related to the population mixture theory, which states that the ancestral north Indians are believed to be descended from western Eurasians, whereas the ancestral south Indians were not related to any group outside India (34). In a population genetic analysis of the Malayalam-speaking population from Kerala, it has been elucidated that the Kerala population is genetically distinct from other Indian populations (35). This has further been demonstrated in another extensive study with PCA and population stratification plots, distinguishing South Indian and North Indian populations using autosomal and mitochondrial markers (36). Moreover, the allele frequency differences between groups in India are larger than in Europe, suggesting strong founder effects whose signatures have been maintained for thousands of years owing to endogamy (37).

The seizure presentation seemed to be higher in our study cohort when compared to East Asian cohorts. This may reflect the referral bias and could be related to the greater proportion of pediatric patients (61%) among whom the seizure is a common clinical presentation than in adults. We did not observe any significant difference between *RNF213* and wild genotype related to the severity of the staging of the disease and bilateral involvement, as reported previously (5, 25).

The patients with *RNF213* carriers had greater scores of periventricular anastomoses. Xue et al. previously showed a remarkable extension of periventricular anastomosis vessels in patients with *RNF213* variants, and subsequently showed the higher expression of *RNF213* in human brain regions in the periventricular area (25).

Fukushima et al. described the phenotypic differences between the homozygous and heterozygous carriers, which are postulated to be related to the gene dosage effect (38). The penetrance of Moyamoya disease in homozygotes is found to be more than 78%, whereas in heterozygotes, it is 0.33–0.67% (39). However, we did not observe any significant difference in clinical presentation or angiographic features or severity of the disease between homozygous and heterozygous carriers.

The study showed significant differences in phenotypic features between *RNF213* known and rare/novel variants. The findings from this study are significant for understanding disease susceptibility and understanding the disease pathology. Recent evidence demonstrates that RNF213 p.R4810K is not only a major susceptibility factor for Moyamoya disease, but also plays a vital role in systemic vasculopathy. Yoshimoto et al. (40) conducted a multi-centric longitudinal study among ischemic stroke or transient ischemic attack (TIA) patients and demonstrated a higher risk of recurrence among RNF213 p.R4810K carriers than non-carriers, and a distinct group of patients at the pre-moyamoya stage. Yoshimoto et al. recently demonstrated the high risk of instant and early reocclusions in RNF213 p.R4810K carriers of ischemic stroke who underwent endovascular therapy for acute occlusion of the intracranial ICA or MCA (41). This suggests the crucial role of genotyping in patients who undergo endovascular therapy and have reocclusion, which is an area of future research.

The study has identified two likely pathogenic variants (p.Arg4810Lys, p.Asp4013Asn) associated with Moyamoya disease according to ACMG criteria: one is an East Asian founder variant, and the other is a Caucasian variant. However, the individual output scores of in silico tools were found to be discordant. In silico pathogenicity tools use different prediction algorithms, and enrichment and updating of tools. Therefore, these discordant outputs emerge from the maturity of the data.

The prospective design of the study enabled us to examine the detailed family history (up to the third generation) of all enrolled patients. The study has not included the secondary Moyamoya cases, and hence, the genetic data truly reflects the specific gene mutations that are associated with the disease. The comprehensive genetic analysis using whole-exome sequencing helped to reveal all susceptibility genes in addition to *RNF213*. The study had certain limitations. As a single-center study conducted at a tertiary referral hospital, the findings may be subject to selection and referral bias, limiting their generalizability. In addition, the proportion of patients enrolled from northern states is relatively small. The small sample size of the study limited the statistical power to detect significant genotype—phenotype correlations and restricted further subgroup analysis. The parental trio sequencing data were not included in this analysis, which could have added more strength to the variant pathogenicity and the inheritance pattern. In addition, the lack of a matched control group limited a comparison of variant frequencies with population-specific polymorphisms.

## 5 Conclusion

The study expands the current understanding of the genetic architecture of Moyamoya disease in the Indian population by demonstrating the RNF213 variant spectrum and its genotype–phenotype correlation. The study accounts for the disparities in the expression of RNF213 p.R4810K and non- p.R4810K variants across north and south Indian regions. The five novel RNF213 variants identified in this study may have important clinical significance in disease pathology and may require further functional validation. The distinct phenotypic profile expressed by p.R4810K and non- p.R4810K variants provides further insights into the role of these variants in different signaling cascades and the pathophysiology of Moyamoya disease. The findings illustrate the need for genetic counseling and cascade screening, particularly in families with a history of Moyamoya disease or early-onset cerebrovascular disease.

## Data Availability

The raw data supporting the conclusions of this article will be made available by the authors, without undue reservation.

## Supplementary material

SUPPLEMENTARY TABLE 1 Description of RNF213 variants identified in the study.

SUPPLEMENTARY Table 2 FoldX-based prediction of changes in protein stability (ΔΔG; total energy) for the five novel RNF213 variants.

SUPPLEMENTARY Table 3 Comparison of the RNF213 variant spectrum and the associated phenotypic features across East Asian, South-East Asian, Caucasian and the present Indian cohort of Moyamoya patients.

SUPPLEMENTARY Table 4 Additional genes associated with Moyamoya disease.

SUPPLEMENTARY Table 5 Comparison of phenotypic features among RNF213 variants of likely pathogenic, uncertain significance and wild type.

SUPPLEMENTARY Figure 1 Pedigrees of RNF213 p.R4810K-positive moyamoya disease families with a positive family history. Legend: A positive family history was present in 4% of the total cohort. Family A included two affected members, a father and daughter, while Families B and C each included one affected member, for a total of four affected patients. Circles indicate females, squares indicate males, and arrowheads mark the proband. A diagonal line through a symbol denotes a deceased individual. Filled symbols indicate affected individuals, and open symbols indicate unaffected individuals.

SUPPLEMENTARY Figure 2 Multiple sequence alignment data of identified RNF213 variants.

SUPPLEMENTARY Figure 3 Structural mapping of the novel RNF213 variants on the hexameric RNF213 structure (PDB ID: 9JW1). Legend: The cryo-EM structure of the RNF213 hexamer (PDB ID: 9JW1) was visualized using PyMOL v3.1.8. (A) Hexameric RNF213 structure with all five novel variants (Q4029R, D4003V, L4117P, Q681E, P2875S) highlighted. (B) Enlarged view of the RING domain showing the three novel variants highlighted in green (Q4029R), magenta (D4003V), and yellow (L4117P).
