# Supplementary material for: Identification and genotype–phenotype correlation of RNF213 p.R4810K and non- p.R4810K variants in Moyamoya disease in an Indian cohort
Source: Front Neurol. 2026 Sep 17;17:1881123. doi: 10.3389/fneur.2026.1881123 (PMC13627448; doi:10.3389/fneur.2026.1881123)
Supplement: Supplementary file 1 [file Table_2.DOCX]

**Supplementary Table 2: FoldX-based prediction of changes in protein stability (ΔΔG; total energy) for the five novel RNF213 variants.**

| **Variant** | **Chain** | **FoldX ΔΔG kcal/mol** | **Effect** |
| --- | --- | --- | --- |
| Q681E | A | 0.377 | Likely neutral |
| P2875S | A | 1.630 | Moderately destabilizing |
| L4117P | A | 4.863 | Strongly destabilizing |
| Q4029R | A | -0.405 | Stabilizing |
| D4003V | A | 1.66481 | Moderately destabilizing |
