# Supplementary material for: Identification and genotype–phenotype correlation of RNF213 p.R4810K and non- p.R4810K variants in Moyamoya disease in an Indian cohort
Source: Front Neurol. 2026 Sep 17;17:1881123. doi: 10.3389/fneur.2026.1881123 (PMC13627448; doi:10.3389/fneur.2026.1881123)
Supplement: Supplementary file 2 [file Table_3.DOCX]

**Supplementary Table 3: Comparison of the *RNF213* variant spectrum and the associated phenotypic features across East Asian, South-East Asian, Caucasian and the present Indian cohort of Moyamoya patients**

|  | **East Asian cohorts (Japan, Korea, China)** | **Southeast Asian cohorts**  **(Vietnam, Philippines)** | **European/American cohorts** | **Indian cohort (present study)** |
| --- | --- | --- | --- | --- |
|  | **Genetic variants** | | | |
| Most common RNF213 variant | p.R4810K (c.14429G>A) is the most common (1)(2)(3)(4) | p.R4810K (c.14429G>A) is absent or rare (4) | p.R4810K is rarely detected.  Rare RNF213 missense variants are more common (examples, p.D4013N, p. R4019C, p.R4062Q, p.R3922Q etc.) (5) | p.R4810K is less common than in East Asians.  Rare RNF213 missense variants are more common (examples, p.D4013N, p.E2191K, p.R4062Q, p.K3982R, p.V3933M, p.R4958Q, etc.) |
| Frequency of RNF213 p.R4810K variant | 70-90% (6) | Absent or rare (7) | Absent or rare (5) | 7% |
|  | **Phenotypic features** | | | |
| Age at onset | Bimodal- peaks around ages 5-9 and 35-39 (8) | Bimodal- childhood and adult onset (7) | Mostly adults, 18 to 44 years of age (9) | Bimodal-childhood and adult onset |
| Female to male ratio | 1.9:1 (10) | 1.6:1 (7) | 4.5:1 (11) | Nearly equal distribution |
| Clinical presentation | Ischemic events predominate in children; adults present with ischemia or intracranial haemorrhage (10) | Ischemic events predominate over TIAs and intracranial haemorrhage (7) | Ischemic stroke and TIAs predominate; haemorrhage is less common (12)(11) | Ischemic stroke and TIAs predominate; haemorrhage is less common |
| Familial cases | 11% to 12% (10) | Rare or absent (7) | Rare or absent (11)(13) | 4% |

RNF213: Ring Finger 213, TIA: Transient ischemic attack

**References**

1.	Liu W, Morito D, Takashima S, Mineharu Y, Kobayashi H, Hitomi T, et al. Identification of RNF213 as a susceptibility gene for moyamoya disease and its possible role in vascular development. PLoS One. 2011;6(7).

2.	Liu W, Hitomi T, Kobayashi H, Harada KH, Koizumi A. Distribution of Moyamoya Disease Susceptibility Polymorphism p.R4810K in RNF213 in East and Southeast Asian Populations. Neurol Med Chir (Tokyo). 2012;52(5):299–303.

3.	Jang MA, Chung JW, Yeon JY, Kim JS, Hong SC, Bang OY, et al. Frequency and significance of rare RNF213 variants in patients with adult moyamoya disease. PLoS One. 2017;12(6):1–9.

4.	Wang Y, Zhang Z, Wei L, Zhang Q, Zou Z. Predictive role of heterozygous p . R4810K of RNF213 in the phenotype of Chinese moyamoya disease. 2020;0(81573240):1–10.

5.	Koizumi A, Kobayashi H, Hitomi T, Harada KH. A new horizon of moyamoya disease and associated health risks explored through RNF213. 2016;55–70.

6.	Duan L, Wei L, Tian Y, Zhang Z, Hu P, Wei Q, et al. Novel susceptibility loci for moyamoya disease revealed by a genome-wide association study. Stroke. 2018;49(1):11–8.

7.	Strunk D, Bauer P, Keyvani K, Diehl RR, Veltkamp R, Berlit P, et al. Moyamoya disease in Southeast Asians: genetic and autopsy data, new cases, systematic review, and meta-analysis of all patients from the literature. J Neurol [Internet]. 2024;271(6):3328–39.

8.	Baba T, Houkin K, Kuroda S. Novel epidemiological features of moyamoya disease. J Neurol Neurosurg Psychiatry. 2008;79(8):900–4.

9.	Miller R, Anikpezie N, Hoffman H, Bako AT, Patel SD, Sahoo A, et al. Demographic Disparities in the Incidence of Moyamoya Angiopathy in the United States. Neurology. 2025;105(5):7–11.

10.	Sato Y, Kazumata K, Nakatani E, Houkin K, Kanatani Y. Characteristics of Moyamoya Disease Based on National Registry Data in Japan. Stroke. 2019;50(8):1973–80.

11.	Saarela M, Mustanoja S, Pekkola J, Tyni T, Hernesniemi J, Kivipelto L, et al. Moyamoya vasculopathy – Patient demographics and characteristics in the Finnish population. Int J Stroke. 2017;12(1):90–5.

12.	Birkeland P. Moyamoya disease beyond Japan and East Asia. Br J Neurosurg [Internet]. 2026;0(0):1–9. Available from: https://doi.org/10.1080/02688697.2026.2634389

13.	Kleinloog R, Regli L, Rinkel GJE, Klijn CJM. Regional differences in incidence and patient characteristics of moyamoya disease: A systematic review. J Neurol Neurosurg Psychiatry. 2012;83(5):531–6.
