# Supplementary material for: Identification and genotype–phenotype correlation of RNF213 p.R4810K and non- p.R4810K variants in Moyamoya disease in an Indian cohort
Source: Front Neurol. 2026 Sep 17;17:1881123. doi: 10.3389/fneur.2026.1881123 (PMC13627448; doi:10.3389/fneur.2026.1881123)
Supplement: Supplementary file 3 [file Table_5.docx]

**Supplementary Table 5: Comparison of phenotypic features among RNF213 variants of likely pathogenic, uncertain significance and wild type**

| Characteristics | RNF213 wild type (n= 74) | RNF213 Variants present (n= 24) | | P Value* |
| --- | --- | --- | --- | --- |
|  |  | VUS (n= 16) | Likely pathogenic (n= 8) |  |
| Age at symptoms onset in years, median (IQR) | 21.0 (28.0) | 17.0 (23.0) | 21.0 (47.1) | 0.382 |
| Age at diagnosis in years, median (IQR) | 23.0 (28.0) | 17.0 (28.0) | 31.5 (36.8) | 0.237 |
| Gender, n (%)   - Males - Females | 32 (43.2%)  42 (56.8%) | 11 (68.8%)  5 (31.3%) | 3 (37.5%)  5 (62.5%) | 0.258 |
| Domicile state, n (%)   - Kerala - Tamil Nadu - West Bengal - Madhya Pradesh - Andhra Pradesh - Maharashtra - Gujarat - Rajasthan - Andaman - Uttar Pradesh | 29 (39.2%)  35 (47.3%)  0 (0)  2 (2.7%)  2 (2.7%)  2 (2.7%)  1(1.4%)  1(1.4%)  1(1.4%)  1(1.4%) | 6 (37.5%)  8 (50%)  1 (6.3%)  0 (0)  0 (0)  0 (0)  0 (0)  1 (6.3)  0 (0)  0 (0) | 0 (0)  3 (37.5%)  5 (62.5%)  0 (0)  0 (0)  0 (0)  0 (0)  0 (0)  0 (0)  0 (0) | **0.012** |
| Domicile state grouping, n (%)   - South Indian states (Kerala, Tamil Nadu, Andhra Pradesh) - North, west and east states | 66 (89.2%)  8 (10.8%) | 14 (87.5%)  2 (12.5%) | 3 (37.5%)  5 (62.5%) | **0.012** |
| Initial clinical presentation- Seizure, n (%)   - No - Yes | 54 (73%)  20 (27%) | 8 (50%)  8 (50%) | 3 (37.5%)  5 (62.5%) | 0.086 |
| Family history of Moyamoya disease, n (%)   - No - Yes | 74 (100%)  0 (0) | 16 (100%)  0 (0) | 4 (50%)  4 (50%) | **0.0002** |
| Posterior cerebral artery involvement, n (%)   - No - Yes | 59 (80.8%)  14 (19.2%) | 15 (93.8%)  1 (6.3%) | 5 (62.5%)  3 (37.5%) | 0.237 |
| Leptomeningeal collaterals, n (%)   - No - Yes | 1 (1.4%)  72 (98.6%) | 0 (0)  16 (100%) | 0 (0)  8 (100%) | 1.000 |
| Suzuki staging, n (%)   - Stages -2 - Stage 3-6 | 9 (12.3%)  64 (87.7%) | 4 (25%)  12 (75%) | 0 (0)  8 (100%) | 0.287 |
| Periventricular anastomosis, n (%)   - 0 - 1 - 2 - 3 - 4 - 5 - 6 | 4 (5.4%)  16 (21.6%)  18 (24.3%)  17 (23%)  10 (13.5%)  5 (6.8%)  2 (2.7%) | 0 (0)  4 (25%)  5 (31.3%)  0 (0)  5 (31.3%)  1 (6.3%)  1 (6.3%) | 1 (12.5%)  0 (0)  0 (0)  1 (12.5%)  1 (12.5%)  2 (25%)  3 (37.5%) | **0.019** |

RNF: Ring Finger Protein, VUS: Variant of uncertain significance, SD: Standard deviation, IQR: Interquartile range, *Benjamini-Hochberg procedure
